# Gist Perception of Image Composition in Abstract Artworks

**DOI:** 10.1177/2041669518780797

**Published:** 2018-06-13

**Authors:** Kana Schwabe, Claudia Menzel, Caitlin Mullin, Johan Wagemans, Christoph Redies

**Affiliations:** Experimental Aesthetics Group, Institute of Anatomy I, University of Jena School of Medicine, Germany; Laboratory of Experimental Psychology, Brain & Cognition, University of Leuven (KU Leuven), Belgium; Experimental Aesthetics Group, Institute of Anatomy I, University of Jena School of Medicine, Germany; Laboratory of Experimental Psychology, Brain & Cognition, University of Leuven (KU Leuven), Belgium

**Keywords:** statistical image properties, gist perception, aesthetics, abstract art, aesthetic rating

## Abstract

Most recent studies in experimental aesthetics have focused on the cognitive processing of visual artworks. In contrast, the perception of formal compositional features of artworks has been studied less extensively. Here, we investigated whether fast and automatic processing of artistic image composition can lead to a stable and consistent aesthetic evaluation when cognitive processing is minimized or absent. To this aim, we compared aesthetic ratings on abstract artworks and their shuffled counterparts in a gist experiment. Results show that exposure times as short as 50 ms suffice for the participants to reach a stable and consistent rating on how *ordered* and *harmonious* the abstract stimuli were. Moreover, the rating scores for the 50 ms exposure time exhibited similar dependencies on image type and self-similarity and a similar pattern of correlations between different rating terms, as the rating scores for the long exposure time (3,000 ms). Ratings were less consistent for the term *interesting* and inconsistent for the term *pleasing*. Our results are compatible with a model of aesthetic experience, in which the early perceptual processing of the formal aspects of visual artworks can lead to a consistent aesthetic judgment, even if there is no cognitive contribution to this judgment.

## Introduction

How much time do human beholders need to appreciate a visual artwork and to form an aesthetic judgment upon it? A comprehensive mental analysis of an artwork’s diverse aesthetic aspects can take up to several minutes. Just consider how long it would take, for example, to contemplate the artistic intentions of a contemporary work of art, why it should be considered novel, the particular mode of presentation at an artistic event, and so on (Brieber, Nadal, Leder, & Rosenberg, 2014; Carbon, 2017; Danto, 1981; Dickie, 1974; Leder, Belke, Oeberst, & Augustin, 2004). Cognitive evaluation requires attention to various details of the artwork, retrieval of information from memory about previous exposure to similar artworks, the integration of this information with art-historical knowledge and other explicit issues that are relevant to an aesthetic experience (Bullot & Reber, 2013; Conway & Rehding, 2013; Pearce et al., 2016). In addition, cycles of feed forward and feedback signaling among these different processes can affect the time course (Belke, Leder, & Carbon, 2015; Leder et al., 2004; Pelowski, Markey, Forster, Gerger, & Leder, 2017).

However, not all processing of complex visual stimuli requires processing of explicit cognitive information. For example, it is well established that human observers can capture the essential visual attributes (i.e., the general meaning) of a scene automatically with just a brief glance (*gist perception*), mainly from the coarse image structure that is transferred to higher visual centers by low-spatial frequencies (Fei-Fei, Iyer, Koch, & Perona, 2007; Oliva, 2005; Oliva & Torralba, 2006). The rapidly produced scene gist is thought to rely on an imprecise representation, in which global relations between elements are maintained but the identity of the local detail is lost (Bachmann & Vipper, 1983; Greene & Oliva, 2009; Morrison & Schyns, 2001; Sampanes, Tseng, & Bridgeman, 2008). Experimental evidence suggests that the low-spatial frequency information of an image is extracted first, followed by recurrent feedback signals that trigger the extraction of fine details of a scene and facilitate object recognition (Bar et al., 2006). In the present study, we asked whether some aspects of the aesthetic evaluation of visual artworks can be perceived by fast and automatic processing as well, similar to gist perception of real-world visual scenes.

Many current models agree that aesthetic experience rests on three pillars: perception, cognition, and emotion (Chatterjee & Vartanian, 2014; Graf & Landwehr, 2015; Leder et al., 2004; Markovic, 2012; Pearce et al., 2016; Redies, 2015; Wagemans, 2011). Cognitive and emotional aspects of artworks have been the subject of many previous investigations in the field of empirical (neuro-)aesthetics (for a review, see Pearce et al., 2016). With respect to perception, there is converging evidence that large sets of artworks from different cultural backgrounds are characterized by specific statistical image properties (SIPs; Brachmann, Barth, & Redies, 2017; Braun, Amirshahi, Denzler, & Redies, 2013; Graham & Field, 2007, 2008; Graham & Redies, 2010; Mather, 2014; Redies, 2015; Redies, Brachmann, & Wagemans, 2017). It has been speculated that the perceptual processing of these properties contributes to the “visual rightness” (Arnheim, 1954) and “good composition” (P. J. Locher, Stappers, & Overbeeke, 1999) of aesthetic images and triggers attributes of aesthetic experience that are universal across cultures and artistic styles (Redies, 2015; Redies et al., 2017). Specific aspects of image composition, like complexity, symmetry, balance, or fractality, can be manipulated easily in simple geometrical patterns and have been the subject of several studies in the past (e.g., Jacobsen, 2004; Spehar, Walker, & Taylor, 2016; Taylor, Spehar, Van Donkelaar, & Hagerhall, 2011; Wilson & Chatterjee, 2005). In contrast, the perception of formal composition in complex artworks has been studied less extensively (Götz, Borisy, Lynn, & Eysenck, 1979; P. Locher, Overbeeke, & Stappers, 2005; McManus, Cheema, & Stoker, 1993), possibly because it is difficult to design visual stimuli that differ in their form only, but not in any of the explicit features that are subject to cognitive processing. Also, it is unclear whether the perceptual processing of image composition alone can lead to an aesthetic evaluation of a visual artwork, when cognitive processing of semantic (explicit) image content and context is minimized or absent, as proposed by Redies (2015). A careful analysis of the aesthetics literature, however, suggests that formal aspects of composition like balance and order play important roles (Wagemans, 2017). Therefore, in the present work, we studied whether fast and automatic processing of image composition can lead to a stable and consistent aesthetic evaluation under conditions that minimize cognitive processing.

**Figure 1.**
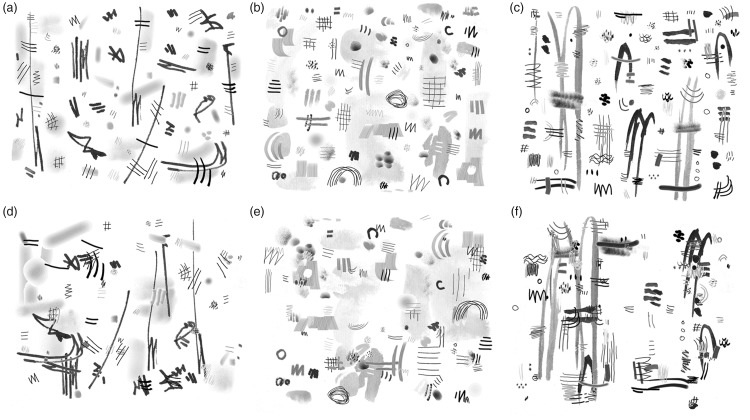
Examples of the stimuli used in the experiment. The pictorial elements of the original images (a–c) were shuffled to create images that lack an artistic composition (d–f). Reproduced with permission. © Christoph Redies, 2017.

In our experiment, we used a set of 20 original abstract artworks that were generated previously by one of the authors (C.R.) and digitized to enable computer-assisted manipulations (Redies, Brachmann, & Hayn-Leichsenring, 2014). Each drawing consisted of 52 to 127 abstract pictorial elements (patches, lines and dots, or small groups thereof), which were arranged by the artist so that the result satisfied his (unspecified) aesthetic criteria. Examples of the drawings are displayed in Figure 1(a) to (c). In the present study, we minimized the influence of (explicit) cognitive information on the aesthetic rating of the images by adopting the following four strategies:

1. *Short presentation times.* Images were presented very briefly, immediately followed by a visual mask. Short presentation times largely prevent the recognition of fine detail in the images, thereby decreasing the recognition and cognitive processing of image detail that takes place later (Bachmann & Vipper, 1983). Several previous studies have shown that particular aspects of the perceptual evaluation of visual artworks or nonart images can be accomplished when the images are displayed rapidly (for a review, see P. Locher, 2015). For example, it has been shown that the attractiveness of faces can be perceived with 13 ms exposure times (Olson & Marshuetz, 2005). Cupchik and Berlyne (1979) demonstrated that participants were able to discriminate properties such as *unity* or *order* in images of representational paintings and artificial patterns after a short glance of only 50 ms (termed ‘painting gist’ by P. Locher, 2015). Differences in the pictorial balance among paintings are detected intuitively and rapidly within 100 ms (P. Locher & Nagy, 1996). Recently, Mullin, Hayn-Leichsenring, Redies, and Wagemans (2017) asked whether the automatic representation of scene gist allows for an aesthetic impression of our environment that is stable and consistent. The authors compared aesthetic judgments on natural scenes with urban and indoor scenes for rapid (50 ms) and unlimited exposures. Their results suggest that aesthetic responses can be extracted rapidly, consistently, and automatically with just a glance at the scenes. Consistent with this, Verhavert, Wagemans, and Augustin (2018) demonstrated that three aspects of aesthetic experience (beauty, specialness, and impressiveness) can be induced by very brief glances (30–50 ms) at artworks of diverse styles. However, it remained unclear whether these judgments were based on sensory visual information (e.g., SIPs) or on more cognitively driven processing (e.g., collative properties, artistic style, or image content).
2. *Abstract artworks*. We used abstract images, which, by definition, are devoid of explicit content that would enable cognitive processing. Moreover, by studying virtually unknown artworks produced by a single artist with the same artistic technique (brush and computer drawing), we minimized any differences in style or art-historical context between the images, which may have lead to differences in aesthetic judgments. Abstract art has been used in experimental studies previously. For example, P. Locher et al. (2005) demonstrated that the distribution of colored areas in Mondrian-type abstract images has an effect on perceived balance of the images. McManus et al. (1993) modified the spacing of line composition in some of Mondrian’s artworks. They found that participants preferred the original Mondrians and thus suggested that these artworks encapsulated some recognizable principle of compositional order. Finally, personality traits were shown to affect the preference of participants for specific SIPs in abstract artworks (Mallon, Redies, & Hayn-Leichsenring, 2014).
3. *Shuffling of pictorial elements*. We compared each original drawing (Figure 1(a)–(c)) with a modified version of the same image wherein the position of the pictorial elements in the image was shuffled by a computer program (Figure 1(d)–(f)). By doing so, we destroyed the image composition that was intended by the artist. In other words, the two types of images differed in their form (artistic or nonartistic) but not in their content or in the pictorial elements. In a previous study (Redies et al., 2014), we showed that the shuffling procedure decreased self-similarity in the images. Our measure of self-similarity reflects how similar the histograms of gradient orientations are in parts of an image compared to the histogram of the entire image. In general, different types of artworks exhibit intermediate to high values of this SIP (Amirshahi, Koch, Denzler, & Redies, 2012; Braun et al., 2013). In the current image set, participants tended to rate the original (artistic) drawings as more *ordered*, more *harmonious* but less *interesting* than the shuffled versions of the drawings, suggesting that the aesthetic perception of the two types of stimuli was different (Redies et al., 2014).
4. *Rating terms*. In aesthetic research, a variety of rating terms have been used, sometimes with different or even opposing experimental results (Augustin, Wagemans, & Carbon, 2012; Cupchik & Berlyne, 1979; Cupchik & Gebotys, 1990; Jacobsen, 2004; Redies et al., 2014; Verhavert et al., 2018). The different terms, which can have positive or negative valence (Augustin et al., 2012), relate to different concepts underlying aesthetic evaluation (Graf & Landwehr, 2015; Markovic, 2012). For example, the terms *ordered* and *harmonious* relate mostly to image structure (Redies et al., 2014), whereas the term *lovely* relates more to affective tone (Markovic, 2010). The usage of some terms is used predominantly for artworks (e.g., *wonderful* and *abstract*), while other terms can be applied more generally to both art and nonart images (e.g., *beautiful* and *ugly*; Augustin et al., 2012). Lyssenko, Redies, and Hayn-Leichsenring (2016) described that structure-related terms tend to be associated with different SIPs. In the present rating experiment, we included structure-related terms (*harmonious* and *ordered*), and a term that relates more to affective or cognitive processing (*interesting;* Cupchik & Gebotys, 1990). We also included a more general aesthetic rating term (*pleasing;* Cupchik & Gebotys, 1990). We hypothesized that structure-related terms would be more consistently used at different exposure times than affect-related terms because image composition is possibly detected by automatic and fast processing at the low-level or mid-level visual system (Redies et al., 2014), while formation of interest and pleasure may require broader, cognitive processing, including recurrent feedback (Pelowski et al., 2017).

We asked the following experimental questions:

1. Does the presence or absence of artistic image composition in the abstract stimuli affect their aesthetic evaluation, in the absence of any differences in cognitive cues?
2. Do the aesthetic ratings of abstract images correlate for rapid and long exposure times?
3. Do the aesthetic evaluations resemble each other for rapid and long exposure times? Specifically, are the correlations between the rating terms themselves and between the rating terms and the SIPs similar for rapid and long exposure times?

## Methods

### Participants

The study included 105 participants (29 males, 75 females, and 1 without indication of gender) who reported normal or corrected-to-normal vision. Two participants who gave monotonous responses without any variations were excluded from the analysis (one participant from the 17 ms group and one from the group with the unlimited exposure time). Of the remaining participants, 90 participants were right-handed, 9 left-handed, 3 ambidextrous, and 1 of unknown handedness. All participants were nonexperts in the field of art. However, they varied in their interest in art from *little* (2 on a scale from 1 to 7) to *very high* (7 on the same scale), and they reported between zero (7 participants) to more than three visits to art museums per year (12 participants).

Forty participants were tested in Leuven, Belgium (15 males, 24 females, 1 unspecified; mean age of 27, range 19–53 years of age) and 65 in Jena, Germany (14 males, 51 females; mean age of 24, range 20–30 years of age). The group of participants in Leuven consisted mainly of psychology students and employees of the Psychology Department, whereas, in Jena, medical students were recruited mostly. Participants received credit points or financial compensation for taking part in the experiment.

The study was conducted in accordance with the ethical principles specified in the World Medical Association Declaration of Helsinki and approved by the Ethics Committees of the KU Leuven and Jena University Hospital (approval number 4808-05/16). All participants gave their written informed consent prior to their participation in the experiment.

### Stimuli

Stimuli consisted of 20 images of grayscale abstract drawings produced by one of the authors (C.R.) and 20 shuffled versions of these drawings. The generation of the images has been described in detail in a previous study (Redies et al., 2014). In brief, each drawing consisted of 52 to 127 isolated pictorial elements on a white background. The first pictorial elements were created with a soft brush in black ink on Japanese rice paper and scanned to obtain a digitized version. Then, other pictorial elements were added at separate levels with a computer drawing program, which simulated artistic materials like brushes, pencils, and so on. The artist did not follow any explicit rules and did not introduce any semantic meaning (e.g., objects or scenery) when drawing and arranging the elements. He finished off when he reached an image composition that satisfied his unspecified, subjective aesthetic criteria.

The generation of the drawings in digitized form allowed moving the position of each pictorial element independently in the final versions of the drawings. From each drawing, another image was generated by shuffling and placing the pictorial elements at randomized positions in the images by the computer. As a result, each drawing was available for testing in the original version and a shuffled version (40 images in total). The final size of the images was 2,048 × 2,048 pixels, which included a narrow white frame of variable size (60–170 pixels on either side) to standardize the aspect ratio. For the image calculations (see later), images were cropped in order to remove the white frame around them. For presentation on the screen, images were downscaled to a size of 1,200 × 900 pixels.

### Statistical Image Properties (SIPs)

SIPs were determined for each of the 40 images. First, all images were reduced in size to 100,000 pixels by bicubic interpolation and isotropic scaling. For every single image, three image properties (self-similarity, complexity, and anisotropy) were then derived from *h*istograms of *o*riented luminance *g*radients (HOG descriptors; Dalal & Triggs, 2005), as described in detail before (Amirshahi et al., 2012; Braun et al., 2013; Redies, Amirshahi, Koch, & Denzler, 2012).

Briefly, self-similarity was calculated by generating HOG descriptors for each image at consecutive levels of a pyramid (Bosch, Zisserman, & Munoz, 2007). We obtained histograms for 16 equally sized bins covering the full circle (Redies et al., 2012). The HOG descriptor was calculated first at the ground level for the entire image (Level 0). The image was then divided into four equally sized rectangles (Level 1). Each section at Level 1 was divided again into four rectangles to generate the next level of the pyramid, and so on. Level 2 thus consisted of 16 sections and Level 3 of 64 sections. For each section at a given level, HOG descriptors were calculated. To obtain a measure of self-similarity, we compared the HOG descriptors at different levels of an image pyramid with the ground level HOG descriptor (Amirshahi et al., 2012; Redies et al., 2012). Self-similarity was defined as the mean self-similarity value for Levels 1 to 3 of the pyramid. A value close to 1 indicates nearly complete self-similarity and a value close to 0 indicates minimal self-similarity. High self-similarity thus implies that subsections of an image exhibit a pattern of oriented luminance gradients which resembles the pattern of the entire image.

Anisotropy was defined as the variance of the luminance gradient strengths across the 16 orientation bins at Level 3 of the HOG pyramid, as described before (Redies et al., 2012). If anisotropy is low, the luminance gradients in the image are homogenously distributed across all orientations of a full circle. A value close to 0 implies an almost uniform distribution across orientations. High anisotropy implies that gradients for some orientation bins are stronger than for others, for example, gradient strength differs across orientations.

As a measure that relates to the subjective complexity of images, we determined the density of oriented gradients (Redies et al., 2012). This measure was defined as the sum of the strengths of all luminance gradients in the entire image.

Traditional artworks of Western provenance possess an intermediate to high degree of self-similarity, low anisotropy, and intermediate complexity, compared to many types of (nonart) image categories (Braun et al., 2013; Redies et al., 2012).

### Procedure

The experiment was developed and first carried out in Leuven to be continued in Jena. Language had to be adapted accordingly in the preexperimental questionnaires and for the experiment instructions (Dutch or English in Leuven, and German in Jena). Otherwise, the testing conditions were kept constant in both places. All participants were interviewed regarding difficulties in performing the tasks immediately following the experiment. They reported no difficulties with respect to comprehending the questions and tasks.

Before the experiment, information about gender, age, handedness, and interest and education in the visual arts was obtained by having each participant fill out a questionnaire. Personal interest in art was evaluated on a scale from 1 to 7 (1 = *no interest*, 7 = *high interest*) and also by the number of art museum/gallery/exhibition visits per year. None of the participants reported having received professional training in the fine arts.

The experiment was carried out in a darkened room in front of a white computer screen (EIZO ColorEdge CG241W, resolution: 1,920 × 1,080 pixels, refresh rate: 60 Hz, color settings: RGB). The monitor was calibrated (including gamma linearization) before the start of the experiment with the i1Display Pro device and the i1Profiler software (X-Rite; Grand Rapids, MI). The distance from the chinrest to the monitor was 75 cm. Stimuli were presented at a size of 170 × 127 mm, which corresponds to 12.9° × 9.7° of visual angle.

The participants were randomly assigned to subgroups for each of five different exposure times (17 ms, 50 ms, 200 ms, 3,000 ms, and unlimited). Every subgroup comprised 21 participants. For each participant, the experiment consisted of four blocks, in which the exposure duration was kept constant. In each block, all 40 images (20 original drawings and 20 shuffled images) were presented in a randomized sequence of trials. The participant was asked to rate each image according to one of the four different terms (*harmonious*, *interesting*, *ordered*, and *pleasing*; for a rationale to use these terms, see “Introduction” section). The other terms were used in the other blocks. The order of the four rating terms was randomized for each participant.

**Figure 2.**
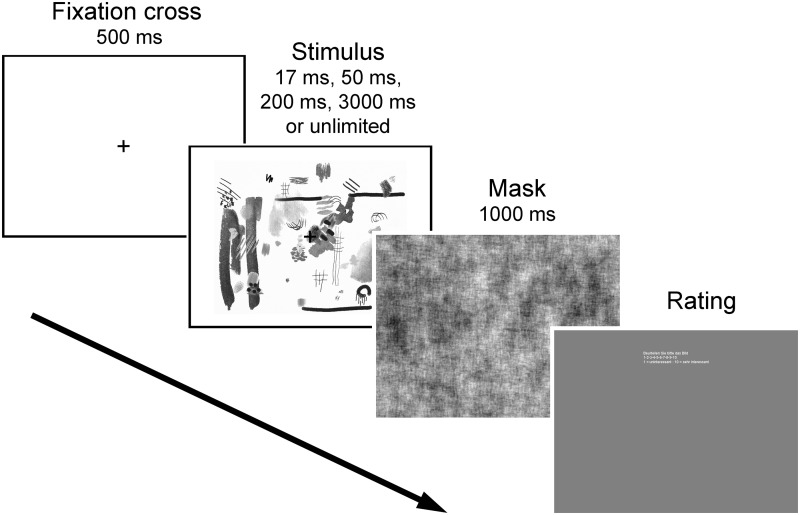
Schematic diagram of the experimental procedure. For unlimited exposure, there was no mask.

Participants received oral instructions before starting the experiment. In particular, they were told that the stimuli were abstract and did not represent or mean anything and that there was no correct or incorrect answer. Participants were asked to give ratings spontaneously according to their gut feeling. Before the first block of the experiment, every participant ran a practice trial with 10 randomly selected stimuli and no specific rating term, in order to get used to the procedure, the exposure time and the stimuli, which were presented in random order during the practice trial. A schematic diagram of the experimental schedule is shown in Figure 2. Each block started with written instructions on the computer screen that indicated which of the rating terms was used in the block. After the self-initiated start of the experiment, a fixation cross appeared for 500 ms prior to the image, which was displayed for the specified time (or until the participant rated the image in the condition with unlimited exposure), immediately followed by a mask for 1,000 ms (except for the condition with unlimited exposure time). The masks were phase-randomized versions of the 40 images used in the experiment and were presented in randomized order. After the mask disappeared, participants were instructed to enter their rating before the experiment continued and the next trial was initiated. The rating was entered on a scale from 1 (e.g., *not harmonious*) to 10 (e.g., *very harmonious*) using the number line of the computer keyboard (10 corresponded to 0 on the keyboard). The computer program recorded the rating score and response time for each trial. After finishing one block of the 40 images, participants were allowed to take a self-paced break before continuing to the next block. The duration of the entire experiment was between 20 and 30 min, depending on the length of individual breaks and the exposure time for each subgroup. Both in Leuven and in Jena, the experiment was performed after carrying out another aesthetic rating experiment, which differed completely from the present experiment in the task and stimuli. The duration of both experiments together did not exceed 1 hr.

### Statistical Analysis

The statistical analysis was conducted in *R* (R Development Core Team, 2017). Four different types of analyses were conducted and are described in the following sections.

First, we assessed the effect of location (Leuven or Jena) by an analysis of variance (ANOVA), with location and exposure time (17 ms, 50 ms, 200 ms, 3,000 ms, or unlimited) as between-subjects factors and image type (original or shuffled) as within-subjects factor. This analysis was performed for each of the four rating terms (*harmonious*, *interesting*, *ordered*, and *pleasing*) separately. Because we did not find any influence of experiment location on the rating scores (see “Results” section), we averaged rating scores over all participants for each exposure time and each image for further analysis.

Second, we analyzed the effect of the three SIPs and the image type on the results of the different rating scales by carrying out multiple linear regression analyses, in which the three SIPs (self-similarity, anisotropy, and complexity) and image type (original or shuffled) were entered as predictors (Model 1). In another model (Model 2), only the three SIPs were entered. The two models were compared by an *R*^2^ difference test to assess whether image type (i.e., artistic or nonartistic image composition) had an effect on the rating scores and on the fit of the model in addition to the effect of the SIPs alone. For each independent variable in each model, we also calculated the standardized regression coefficients β and tested whether this variable predicted the rating scores when the other variables were controlled for (see *p* values for each variable).

Third, to study whether the rating scores were consistent between different exposure times, especially between short and long exposure times, we calculated Pearson correlation coefficients and carried out Fisher *r*-to-*z* transformation. This analysis was performed for each of the four rating terms separately. Specifically, the rating scores for exposure times of 17 ms, 50 ms, 200 ms, and 3,000 ms were correlated with the rating scores for unlimited exposure time, and the rating scores for exposure times of 17 ms, 50 ms, and 200 ms with those for an exposure time of 3,000 ms. Rating scores for the 40 stimuli were averaged for each participant and rating term. In addition, by the use of another ANOVA, we determined the effect of exposure time as between-subjects factor and image type (original or shuffled) as within-subjects factor as well as their interactions on each rating scale.

Fourth, we correlated the rating scores for the different terms with each other to assess how much they depended on each other or were independent. To this aim, Pearson correlation coefficients *r* were calculated and transformed to Fisher *z* scores.

**Figure 3.**
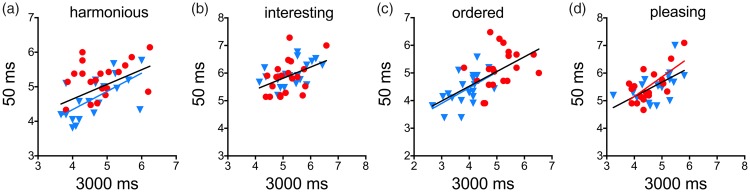
Scatter plots of average rating scores (a, *harmonious*; b, *interesting*; c, *ordered*; and d, *pleasing*) for the exposure times of 50 ms and 3,000 ms (*red dots*, original images; *blue triangles*, shuffled versions). The lines represent significant results from a linear regression analysis (*black* lines, both image types together; *red* line, original images; and *blue* lines, shuffled versions).

**Figure 4.**
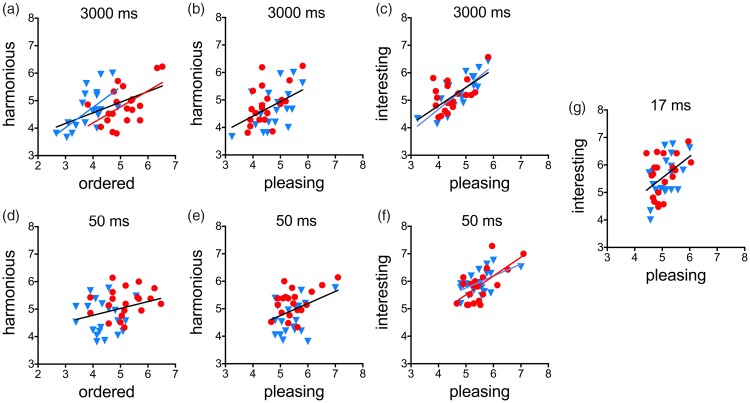
Scatter plots of scores for the different rating scales for exposure times of 3,000 ms (a–c), 50 ms (d–f) and 17 ms (g). *Red dots* indicate original images and *blue triangles* indicate the shuffled versions. The lines represent significant results from a linear regression analysis (*black* lines, both image types together; *red* line, original images; and *blue* lines, shuffled versions).

In all of the correlation analyses, we used two-sided tests and carried out Bonferroni corrections for multiple correlations to avoid alpha-error accumulation. Scatter plots of data that yielded significant correlations are shown in Figures 3 and 4. In these figures, we also show the fitted lines from a linear regression analysis, but only if the regression lines had a slope significantly different from zero (*black* lines, all images; *red* lines, original images; and *blue* lines, shuffled images).

## Results

The ANOVA that included location (Leuven or Jena) as a factor did not reveal any influence of experiment location on the rating scores for the different rating terms and exposure times, respectively. In the following analyses, data from the two locations were therefore grouped together.

At unlimited exposure times, the time interval between stimulus onset and the pressing of the keyboard to register the rating varied to a large degree between participants (mean 2.8 s ± 2.3 standard deviation [*SD*]). Without a fixed exposure time, some of the participants gave their ratings after a relatively short exposure time and then proceeded to the next image. Indeed, the mean response time of a particularly speedy participant was only about 1,000 ms. To reach similar experimental conditions for all participants, we therefore decided to focus the following analyses on the longest fixed exposure time of 3,000 ms.

### Effect of SIPs on the Ratings

First, we studied whether the ratings at short and long exposure times depended in a similar fashion on specific SIPs (see “Introduction” section). Scores were entered into a multiple linear regression analysis, starting with the exposure time of 3,000 ms. We then asked whether the dependencies observed for this long exposure time were stable or changed when exposure times was successively shortened.

Results are shown in Table 1 for 3,000 ms and 200 ms, and in Table 2 for 50 ms and 17 ms exposure time. Two models were considered: In Model 1, the SIPs (self-similarity, anisotropy, and complexity) and image type were entered as predictors. Because a previous study (Redies et al., 2014) showed differences in self-similarity between the image types (original drawings: mean 0.69 ± 0.05 *SD*; shuffled versions: mean 0.57 ± 0.05 *SD*; *p* < .001), we also tested a model with the SIPs, but without image type as an independent variable (Model 2), to remove possible redundancies.

**Table 1.** Results From Multiple Linear Regression Analyses for Exposure Times of 3,000 ms and 200 ms.

	Model 1 (*df*: 4,35)				Model 2 (*df*: 3,36)			
Exposure time	Variable	β	*t* value	*p* value	Variable	β	*t* value	*p* value
3,000 ms	*Harmonious* (*R*^2^_adj_ = .11, *F* = 2.10, *p* = .101)				*Harmonious* (*R*^2^_adj_ = .096, *F* = 2.38, *p* = .086)			
	Self-similarity	0.12	0.36	.718	Self-similarity	0.36	1.38	.176
	Anisotropy	0.23	0.94	.356	Anisotropy	0.25	1.01	.320
	Complexity	−0.39	−2.25	.031	Complexity	−0.42	−2.42	.021
	Image type	−0.27	−1.11	.275				
	*Interesting* (*R*^2^_adj_ = .06, *F* = 0.44, *p* = .775)				*Interesting* (*R*^2^_adj_ = .03, *F* = 0.61, *p* = .614)			
	Self-similarity	−0.33	−0.92	.363	Self-similarity	−0.32	−1.16	.254
	Anisotropy	−0.23	−0.85	.400	Anisotropy	−0.23	−0.86	.395
	Complexity	−0.05	−0.27	.790	Complexity	−0.05	−0.28	.778
	Image type	−0.39	−0.06	.953				
	***Ordered* (*R*^2^_adj_ = .56, *F* = 13.5, *p* < .001)**				***Ordered* (*R*^2^_adj_ = .32, *F* = 7.21, *p* < .001)**			
	Self-similarity	0.27	0.26	.259	Self-similarity	0.94	4.21	<.001
	Anisotropy	0.34	1.15	.057	Anisotropy	0.39	1.83	.076
	Complexity	−0.18	1.97	.150	Complexity	−0.26	−1.71	.095
	Image type	−0.77	−4.55	<.001				
	*Pleasing* (*R*^2^_adj_ = .03, *F* = 1.31, *p* = .286)				*Pleasing* (*R*^2^_adj_ = .055, *F* = 1.75, *p* = .173)			
	Self-similarity	−0.24	−0.68	.500	Self-similarity	−0.17	−0.62	.537
	Anisotropy	0.09	0.37	.716	Anisotropy	0.10	0.40	.696
	Complexity	−0.15	−0.85	.403	Complexity	−0.16	−0.91	.367
	Image type	−0.08	−0.32	.748				
200 ms	***Harmonious* (*R*^2^_adj_ = .30, *F* = 5.14, *p* = .002)**				***Harmonious* (*R*^2^_adj_ = .27, *F* = 5.76, *p* = .003)**			
	Self-similarity	0.48	1.64	.110	Self-similarity	0.78	3.36	.002
	Anisotropy	0.18	0.82	.419	Anisotropy	0.20	0.91	.370
	Complexity	−0.20	−1.29	.207	Complexity	−0.23	−1.49	.144
	Image type	−0.34	−1.59	.120				
	*Interesting* (*R*^2^_adj_ = .06, *F* = 1.58, *p* = .201)				*Interesting* (*R*^2^_adj_ = .08, *F* = 2.12, *p* = .115)			
	Self-similarity	−0.01	−0.03	.973	Self-similarity	−0.09	−0.35	.725
	Anisotropy	0.18	0.70	.488	Anisotropy	0.17	0.69	.497
	Complexity	0.42	2.34	.025	Complexity	0.43	2.44	.020
	Image type	0.09	0.37	.713				
	***Ordered* (*R*^2^_adj_ = .68, *F* = 21.6, *p* < .001)**				***Ordered* (*R*^2^_adj_ = .46, *F* = 11.91, *p* < .001)**			
	Self-similarity	0.16	0.84	.407	Self-similarity	0.82	4.06	<.001
	Anisotropy	0.09	0.58	.569	Anisotropy	0.14	0.71	.484
	Complexity	0.06	0.x56	.580	Complexity	−0.02	−0.12	.904
	Image type	−0.74	−5.10	<.001				
	*Pleasing* (*R*^2^_adj_ = .09, *F* = 1.95, *p* = .124)				*Pleasing* (*R*^2^_adj_ = .11, *F* = 2.67, *p* = .062)			
	Self-similarity	0.22	0.67	.510	Self-similarity	0.20	0.78	.441
	Anisotropy	−0.12	−0.49	.627	Anisotropy	−0.12	−0.51	.615
	Complexity	0.18	1.04	.306	Complexity	0.18	1.08	.287
	Image type	0.03	0.11	.910				

*Note*. Model 1 describes the effect of the SIPs (self-similarity, anisotropy and complexity) and image type as predictors of the rating scores. Model 2 describes the effect of the SIPs only. Significant models are marked in bold letters (*p* < .05).

**Table 2.** Results From Multiple Linear Regression Analyses for Exposure Times of 50 ms and 17 ms.

	Model 1 (*df*: 4,35)				Model 2 (*df*: 3,36)			
Exposure time	Variable	β	*t* value	*p* value	Variable	β	*t* value	*p* value
50 ms	***Harmonious* (*R*^2^_adj_ = .29, *F* = 4.90, *p* = .0030)**				*Harmonious* (*R*^2^_adj_ = .032, *F* = 1.43, *p* = .249)			
	Self-similarity	−0.69	−2.29	.028	Self-similarity	0.02	0.07	.943
	Anisotropy	−0.38	−1.73	.093	Anisotropy	−0.33	−1.27	.212
	Complexity	−0.15	−0.95	.350	Complexity	−0.23	−1.27	.211
	Image type	−0.80	−3.71	<.001				
	*Interesting* (*R*^2^_adj_ = .08, *F* = 0.25, *p* = .909)				*Interesting* (*R*^2^_adj_ = .054, *F* = 0.34, *p* = .798)			
	Self-similarity	−0.15	−0.41	.687	Self-similarity	−0.14	−0.49	.629
	Anisotropy	0.06	0.21	.836	Anisotropy	0.06	0.22	.830
	Complexity	0.11	0.57	.572	Complexity	0.11	0.58	.568
	Image type	−0.01	−0.06	.957				
	***Ordered* (*R*^2^_adj_ = .26, *F* = 4.47, *p* = .0051)**				*Ordered* (*R*^2^_adj_ = .024, *F* = 1.32, *p* = .283)			
	Self-similarity	−0.20	−0.64	.524	Self-similarity	0.49	1.82	.077
	Anisotropy	0.17	0.77	.445	Anisotropy	0.23	0.88	.383
	Complexity	0.00	−0.02	.988	Complexity	−0.08	−0.45	.655
	Image type	−0.78	−3.55	.001				
	*Pleasing* (*R*^2^_adj_ = .04, *F* = 0.65, *p* = .630)				*Pleasing* (*R*^2^_adj_ = .058, *F* = 0.29, *p* = .836)			
	Self-similarity	−0.39	−1.09	.282	Self-similarity	−0.09	−0.33	.743
	Anisotropy	−0.03	−0.12	.904	Anisotropy	−0.01	−0.03	.974
	Complexity	−0.06	−0.31	.759	Complexity	−0.09	−0.49	.625
	Image type	−0.34	−1.32	.197				
17 ms	*Harmonious* (*R*^2^_adj_ = .02, *F* = 0.78, *p* = .546)				*Harmonious* (*R*^2^_adj_ = .003, *F* = 0.96, *p* = .423)			
	Self-similarity	0.14	0.40	.696	Self-similarity	0.27	0.97	.338
	Anisotropy	−0.01	−0.04	.969	Anisotropy	0.00	−0.002	.998
	Complexity	−0.25	−1.34	.190	Complexity	−0.26	−1.44	.158
	Image type	−0.39	−0.55	.588				
	***Interesting* (*R*^2^_adj_ = .19, *F* = 3.35, *p* = .0202)**				***Interesting* (*R*^2^_adj_ = .20, *F* = 4.27, *p* = .011)**			
	Self-similarity	−0.05	−0.16	.872	Self-similarity	0.12	0.48	.635
	Anisotropy	0.44	1.86	.718	Anisotropy	0.45	1.93	.062
	Complexity	0.52	3.16	.0032	Complexity	0.50	3.09	.004
	Image type	−0.19	−0.84	.409				
	***Ordered* (*R*^2^_adj_ = .23, *F* = 3.91, *p* = .0100)**				***Ordered* (*R*^2^_adj_ = .25, *F* = 5.36, *p* = .0037)**			
	Self-similarity	0.53	1.72	.094	Self-similarity	0.57	2.39	.022
	Anisotropy	0.32	1.39	.173	Anisotropy	0.32	1.43	.163
	Complexity	0.30	1.89	.067	Complexity	0.30	1.91	.064
	Image type	−0.04	−0.16	.872				
	***Pleasing* (*R*^2^_adj_ = .21, *F* = 3.64, *p* = .0140)**				***Pleasing* (*R*^2^_adj_ = .22, *F* = 4.61, *p* = .0078)**			
	Self-similarity	−0.44	−1.42	.165	Self-similarity	−0.27	−1.11	.275
	Anisotropy	0.18	0.77	.444	Anisotropy	0.19	0.84	.407
	Complexity	0.58	3.56	.001	Complexity	0.56	3.48	.001
	Image type	−0.20	−0.89	.379				

*Note*. Model 1 describes the effect of the SIPs (self-similarity, anisotropy, and complexity) and image type as predictors of the rating scores. Model 2 describes the effect of the SIPs only. Significant models are marked in bold letters (*p* < .05). SIPs = statistical image properties.

For 3,000 ms (Table 1), both regression models were significant only for the rating scores for *ordered*. Model 1 revealed that image type was the only variable that predicted the rating scores when the other variables were controlled for. In Model 2 (without image type), self-similarity was the strongest predictor. The explained variance (*R*^2^_adj_) was larger for Model 1 than for Model 2 (*R*^2^ difference test [ANOVA]), *F*(35, 36) = 20.7, *p* < .001, suggesting that the effect of image type on the rating can be explained only in part by the difference in self-similarity between the image types.

For 200 ms (Table 1), we obtained a difference between the two models for *ordered*, *F*(35, 36) = 26.0, *p* < .001. Both models also predicted ratings for *harmonious* but did not differ in the percentage of variance predicted, *F*(35, 36) = 2.54, *p* = .12. Again, self-similarity was the strongest predictor in Model 2. For 50 ms (Table 2), Model 1 predicted ratings for *ordered* and *harmonious,* with image type being the strongest predictor in both cases.

Results for 17 ms (Table 2) showed a different pattern of dependencies on the SIPs. In both models, complexity was the only significant predictor for the rating scores of *interesting* and *pleasing* when the other variables were controlled for. The two models predicted a similar amount of variance in both cases, *interesting*: *F*(35, 36) = 0.70, *p* = .41; *pleasing*: *F*(35, 36) = 0.79, *p* = .38, suggesting that the effect was independent of image type. The two models also predicted ratings for *ordered* to a similar degree, *F*(35, 36) = 0.03, *p* = .87, with self-similarity being the strongest predictor.

### Correlations Between the Rating Scores for Short and Long Exposure Times

Next, we asked to what extent the rating scores for a given term were consistent at short and long exposure times and calculated Pearson correlation coefficients, which were transformed to Fisher *z* scores. Tables 3 and 4 list the correlations between the rating scores for the short exposure times (17 ms, 50 ms and 200 ms) and the rating scores for the unlimited and 3,000 ms exposure time, respectively.

**Table 3.** Fisher *z* Scores for the Pearson Correlations Between Mean Rating Scores for Different Short Exposure Times and the Rating Scores for the Long Exposure Time of 3,000 ms (*df* = 38).

Exposure time	*Harmonious*	*Interesting*	*Ordered*	*Pleasing*
17 ms	0.25 (*p* = .122)	0.18 (*p* = .285)	0.19 (*p* = .256)	0.41* (*p* = .013)
50 ms	0.51* (*p = *.002)	0.50* (*p* = .003)	0.75* (*p* = .010)	0.59* (*p* < .001)
200 ms	0.52* (*p = *.005)	0.46* (*p* = .005)	0.84* (*p* = .001)	0.12 (*p* = .480)

* *p* < .0167 (Bonferroni-corrected).

**Table 4.** Fisher *z* Scores for the Pearson Correlations Between Mean Rating Scores for Different Fixed Exposure Times and the Rating Scores for Unlimited Exposure Time (*df* = 38).

Exposure time	*Harmonious*	*Interesting*	*Ordered*	*Pleasing*
17 ms	0.33 (*p* = .042)	0.46* (*p* = .006)	0.34 (*p* = .041)	0.26 (*p* = .112)
50 ms	0.59* (*p* < .001)	0.29 (*p* = .079)	0.96* (*p* < .001)	0.46* (*p* = .006)
200 ms	0.85* (*p* < .001)	0.66 (*p* = .094)	0.99* (*p* < .001)	0.64* (*p* < .001)
3,000 ms	0.75* (*p* < .001)	0.28 (*p* = .092)	1.29* (*p* < .001)	0.34 (*p* = .040)

* *p* < .0125 (Bonferroni-corrected).

The results for the 40 images (averaged over participants) do not show any correlations between the rating scores for the exposure time of 3,000 ms and for the shortest exposure time of 17 ms (Table 3). For the exposure time of 50 ms, scores for all four rating terms yielded significant correlations with the results for the 3,000 ms exposure time. The correlations were strongest for the rating term *ordered*, followed by *pleasing*. For the exposure time of 200 ms, the results were also correlated, except for the term *pleasing*. Again, the strongest correlation was obtained for the term *ordered*.

Results were roughly similar when rating scores for short exposure times were compared to those for the unlimited exposure time. In particular, for *harmonious* and *ordered*, correlations were obtained for exposure times of 50 ms, 200 ms, and 3,000 ms but not for 17 ms. Results were different for *interesting*: Rating scores correlated with those for 17 ms but not with the rating scores for the other exposure times (50 ms, 200 ms and 3,000 ms). For *pleasing*, a correlation was observed for 50 ms and 200 ms but not for 3,000 ms exposure time.

To study the correlations between the rating scores and the interaction with image type in more detail, we analyzed the rating scores for 50 ms and for 3,000 ms, as an example. Figure 3 visualizes the correlations between the rating scores for the two exposure times. For all four rating terms, correlations were significant (cf. Table 3). In addition, significant slopes of the regression lines were also obtained with the shuffled versions for *harmonious* (*r* = .60, *p* = .005; Figure 3(a)) and for *ordered*, respectively (*r* = .61, *p* = .004; Figure 3(c)) and with the original drawings for *pleasing* (*r* = .66, *p* = .002; Figure 3(d)).

The effect of exposure time and image type (original or shuffled) on the rating scores was assessed by an ANOVA. The mean rating scores and post-hoc comparisons for the two image types (original and shuffled) are listed in Table 5. Whereas exposure time did not have an effect on any of the rating scores for either term, effects of image type on the ratings were observed. For *ordered*, *F*(1, 98) = 72.16; *p* < .001; η^2^_p_ = .42, original drawings were rated higher (mean 5.5 ± 1.6 *SD*) than the shuffled versions (mean 4.3 ± 1.2 *SD*). In addition, there was an interaction for *ordered* between exposure time and image type, *F*(4, 98) = 4.01; *p* = .005; η^2^_p_ = .14. For each of the fixed exposure times, the *ordered* ratings differed between the two image types, with higher ratings given to the original (artistic) compositions. We did not find an effect of exposure time on rating scores when image types were tested separately. There was also a difference between image types for *harmonious*, *F*(1, 97) = 17.51; *p* < .001; η^2^_p_ = .15. Here, original drawings were rated higher on average (mean 5.3 ± 1.3 *SD*) than the shuffled versions (mean 4.8 ± 1.1 *SD*). All other effects were not significant.

**Table 5.** Mean Values for the Different Rating Terms.

Exposure time	Original images	Shuffled images	*p* value
*Harmonious* (*ns*)			
17 ms	5.1 (± 1.2 *SD*)	4.9 (± 1.3 *SD*)	
50 ms	5.2 (± 1.2 *SD*)	4.7 (± 1.1 *SD*)	
200 ms	5.8 (± 1.3 *SD*)	5.0 (± 1.1 *SD*)	
3,000 ms	4.8 (± 1.3 *SD*)	4.7 (± 1.0 *SD*)	
*Interesting* (*ns*)			
17 ms	5.7 (± 0.9 *SD*)	5.6 (± 1.0 *SD*)	
50 ms	5.8 (± 1.3 *SD*)	6.0 (± 1.5 *SD*)	
200 ms	5.5 (± 1.3 *SD*)	5.6 (± 0.8 *SD*)	
3,000 ms	5.2 (± 1.4 *SD*)	5.3 (± 1.2 *SD*)	
*Ordered*			
17 ms	5.1 (± 1.1 *SD*)	4.7 (± 0.9 *SD*)	.01
50 ms	5.2 (± 1.6 *SD*)	4.4 (± 1.5 *SD*)	.004
200 ms	6.4 (± 1.3 *SD*)	4.3 (± 1.0 *SD*)	<.001
3,000 ms	5.1 (± 1.9 *SD*)	3.8 (± 1.1 *SD*)	.007
*Pleasing* (*ns*)			
17 ms	5.1 (± 1.1 *SD*)	5.1 (± 1.0 *SD*)	
50 ms	5.5 (± 1.4 *SD*)	5.5 (± 1.6 *SD*)	
200 ms	5.4 (± 1.4 *SD*)	5.1 (± 1.0 *SD*)	
3,000 ms	4.5 (± 1.4 *SD*)	4.7 (± 1.2 *SD*)	

*Note*. The *p* values indicate the significance level of the interactions between exposure time and image type (post-hoc *t* tests). For *F* statistics, see main text. *ns* = interaction between exposure time and image type not significant (analysis of variance).

### Correlations Between the Scores for Different Rating Terms

Next, we asked whether the scores obtained for the different rating terms showed similar relationships between each other for short and long exposure times, respectively. Such similarities would be expected if similar rating criteria were used for short and long exposure times (see “Introduction” section). Therefore, we first calculated the correlations between the rating terms for the longest exposure time (3,000 ms) and then asked in how far similar patterns of correlations were observed for shorter exposure times. Results are summarized in Table 6. The scatter plots in Figure 4 visualize some of the correlations for exposure times of 3,000 ms, 50 ms and 17 ms.

**Table 6.** Fisher *z* Scores for Pearson Correlations Between the Rating Scores for the Four Terms (*Harmonious, Interesting, Ordered*, and *Pleasing*) for the Exposure Times of 3000 ms, 200 ms, 50 ms, and 17 ms.

Rating term	*Harmonious*	*Interesting*	*Ordered*	*Pleasing*
	3000 ms			
*Harmonious*	–	0.34	0.53*	0.55*
*Interesting*	*p* = .040	–	0.08	0.79*
*Ordered*	*p* = .001	*p* = .622	–	0.09
*Pleasing*	*p* = .001	*p* = .004	*p* = .598	–
	200 ms			
*Harmonious*	–	−0.15	0.78*	0.42
*Interesting*	*p* = .371	–	−0.23	0.37
*Ordered*	*p* = .005	*p* = .154	–	0.25
*Pleasing*	*p* = .012	*p* = .027	*p* = .136	–
	50 ms			
*Harmonious*	–	0.002	0.57*	0.42
*Interesting*	*p* = .992	–	−0.24	0.69
*Ordered*	*p* < .001	*p* = .150	–	0.27
*Pleasing*	*p* = .011	*p* = .051	*p* = .103	–
	17 ms			
*Harmonious*	–	−0.02	0.21	0.11
*Interesting*	*p* = .891	–	−0.08	0.49*
*Ordered*	*p* = .202	*p* = .617	–	0.05
*Pleasing*	*p* = .493	*p* = .004	*p* = .772	–

Note: Fisher *z* and corresponding *p* values are above and below the diagonal, respectively. *p < .0083 (Bonferroni-corrected).

For 3,000 ms exposure time, significant positive correlations of similar strength were obtained between the rating term pairs *harmonious*/*ordered* (Figure 4(a)) and *harmonious*/*pleasing* (Figure 4(b)). The correlation for *harmonious*/*ordered* remained significant when each image type was evaluated separately (original images, *r* = .55, *p* = .012, *red* line in Figure 4(a); shuffled images, *r* = .64, *p* = .002, *blue* line). The correlation for *interesting/pleasing* was even stronger and was also obtained for the subset of shuffled images (*r* = .76, *p* < .001, *blue* line in Figure 4(c)).

Results for 200 ms exposure time were similar to results for 3,000 ms exposure time in that a positive correlation was obtained for the rating term pair *harmonious*/*ordered* and a tendency of a positive correlation for *harmonious*/*pleasing* (Table 6). However, there was no correlation for *interesting/pleasing*. For an exposure time of 50 ms, the same pattern of correlations was found (Table 6, Figure 3(d)–(f)). In addition, for *interesting/pleasing*, significant correlations were found when image type was considered separately (original images, *r* = .65, *p* = .002, *red* line in Figure 3(f); shuffled images, *r* = .55, *p* = .012, *blue* line). For 17 ms exposure time, a correlation between results for different rating terms was obtained for *interesting/pleasing* only (Table 6, Figure 4(g)).

In summary, the correlation for the rating term pair *harmonious*/*ordered*, which was observed at the short exposure time (50 ms), persisted at the long exposure time (3,000 ms). The correlation for *interesting/pleasing* was present also for the very short exposure time of 17 ms but was not observed for the exposure times of 200 ms and 50 ms.

## Discussion

The present gist experiment demonstrates that, for the terms *ordered*, *harmonious*, and *interesting,* exposure times as short as 50 ms suffice to reach rating scores that are stable over time and consistent with the rating scores at long exposure times (Tables 3 and 4, Figure 3). For *ordered* and *harmonious*, the rating scores for the 50 ms exposure time exhibited a similar pattern of correlations between the scores of the different ratings terms (Table 6, Figure 4(d)–(f)) and similar dependencies on self-similarity and image type (Table 2), as the rating scores for the long exposure time (3,000 ms; Table 1, Figure 4(a)–(c)). Ratings for very short exposure times (17 ms) resulted in a different and less consistent pattern. Here, rating scores for *interesting* and *pleasing* correlated with each other (Table 6, Figure 4(g)), as they did for long exposure times (3,000 ms), but these rating scores depended more on the complexity than on the self-similarity of the stimuli (Table 2).

### Enhancing the Role of Perceptual Processing in Aesthetic Evaluation

The aim of the present experiment was to investigate whether an aesthetic evaluation of visual artworks is possible under conditions when perceptual mechanisms are enforced, at the expense of cognitive mechanisms (see “Introduction” section). We pursued this aim by using short exposure times and comparing abstract drawings with artistic intent and their shuffled counterparts without artistic intent, thereby largely eliminating differences between the images in depicted content or cultural context, which may have led to differences in cognitive evaluation. With our between-subjects design, we obtained rating scores that were stable at long and short exposure times, down to 50 ms exposure time. We thus conclude that aesthetic judgments based on the perceptual processing of artworks are possible, even if cognitive processing is effectively minimized.

Rating scores were most consistent for the two terms that reflect structural properties of the images (*ordered* and *harmonious*; Tables 3 and 4). For *interesting*, rating scores were also consistent but only for a comparison of the rapid display (50 ms and 200 ms) and 3,000 ms exposure time. Moreover, we did not observe a stable dependency on any SIP for *interesting* (Tables 1 and 2). Although *interesting* ratings have also been associated with the structural complexity of an image (Cupchik & Gebotys, 1990), an affective component has been ascribed to this rating term (Berlyne, 1974; Silvia, 2005). This component might be the reason why the *interesting* ratings show more interindividual variability at short exposure time when compared to the structure-related terms *ordered* and *harmonious.* Rating scores were even less stable for *pleasing*, which mirrors more subjective impressions of the beholder and possibly requires an integration of self-reflective, internal information on personal taste and preferences (Pelowski et al., 2017). In conclusion, as expected, the rating terms that are more clearly associated with image structure (*ordered* and *harmonious*) are more stable with brief exposure times than the two terms that also reflect more affective and subjective aspects of aesthetic judgment (*interesting* and *pleasing*).

### Time Course of Aesthetic Perception of Image Composition

A number of previous studies have investigated the time course of aesthetic perception, in particular for short presentation times that allow only a single glance at an artwork (10–100 ms). For example, Cupchik and Berlyne (1979) studied the perception of descriptive properties in 18 mostly representational paintings and synthetic patterns for viewing times of 50 ms, 500 ms, or 5,000 ms. They asked participants to rate the images along different descriptive scales (e.g., *disorderly/orderly, simple/complex*), including hedonic scales (e.g., *ugly/beautiful, displeasing/pleasing*). Participants in their study were able to discriminate these properties not only at long exposure times, but also after a short display of 50 ms, in particular for such rating terms as *order* and *unity* (Cupchik & Berlyne, 1979). Findings from the present study resemble these and other results (Bachmann & Vipper, 1983; P. Locher, 2015). Another global image property that relates to image structure and can be detected intuitively and rapidly within a single glance (100 ms presentation duration) is pictorial balance (P. Locher & Nagy, 1996). Furthermore, P. Locher, Krupinski, Mello-Thoms, and Nodine (2007) showed that an initial holistic impression of the structural elements and semantic meaning of the paintings can be reached with a single 100 ms glance. Augustin, Leder, Hutzler, and Carbon (2008) investigated the recognition of similarities in content and style of representational artworks with systematically varying presentation times (10 ms, 50 ms, 202 ms, and 3,000 ms). The authors observed effects of style from 50 ms onward, whereas effects of content were present already at 10 ms (Augustin et al., 2008). A follow-up event-related potential study revealed a similar processing sequence of style following content (Augustin, Defranceschi, Fuchs, Carbon, & Hutzler, 2011). The study by Verhavert et al. (2018) confirmed that consistent aesthetic judgments can be reached with a short glance at artworks of different styles. Like in the present study, the authors compared the time course of three different rating terms and found that *impressiveness* judgments require longer exposure times and are less consistent than impressions of *beauty* and *specialness*. Finally, Mullin et al. (2017) showed that gist perception allows for an automatic and stable aesthetic impression to be extracted from real-world images (photographs of exterior and interior scenes). Moreover, the pattern of preferences, which the participants had for the different image types, interacted significantly with the same image property that showed an effect in the present study (i.e., self-similarity; Tables 1 and 2).

The present results confirm and extend these studies. First, we show that human observers reach a stable impression of how *ordered* abstract images with and without artistic intent are when the images are viewed at a short glance. The difference in self-similarity between the image types is one of the features that might contribute to this finding (Table 5). However, because self-similarity explains less variance in the rating scores than image type (see *R*^2^_adj_ values for exposure times of 3,000 ms and 200 ms in Table 1), other unspecified image properties possibly also contribute to the rating of *ordered*. Second, we demonstrate that, at the short and long exposure times, the rating scores for *ordered* and *harmonious* show a similar pattern of correlations with results for the other rating terms (Figure 4, Table 6) and similar dependencies on image properties (Tables 1 and 2). These findings suggest that the rating scores are not only stable when perceptual processing is enforced, but are also based on the same or closely related perceptual mechanisms, even when there is enough time for cognitive processes to set in at the longer exposure times. In a companion study (Menzel, Kovács, Amado, Hayn-Leichsenring, & Redies, 2018), we recorded event-related electrophysiological potentials in response to the same abstract stimuli that were used in the present study and found that differences between the original and shuffled versions are detected automatically by the human visual system.

Intriguingly, rating scores for *pleasing* were less consistent and stable over time, also with respect to their dependency on the image properties. For this term, rating might require more time because it taps into internal representations of individual taste and preference. This result is in line with electrophysiological recordings by Jacobsen and Höfel (2003) who asked participants to rate the beauty and symmetry of simple geometrical patterns. Their findings indicated that aesthetic judgments are mediated by a two-stage process that consists of an initial impression formation at about 300 ms and a deeper aesthetic evaluation at around 600 ms after stimulus onset. The time required for cognitive information processing varies greatly between individuals and for different artworks (Brieber et al., 2014; Heidenreich & Turano, 2011; Smith & Smith, 2001; Tröndle & Tschacher, 2012).

In summary, we provide evidence that fast, automatic, and stable aesthetic evaluations of abstract artworks can be accomplished under conditions when differential cues for cognitive processing are minimized or absent. For the stimuli used in the present study, the aesthetic evaluations depended on a specific SIP (self-similarity), at least in part. As expected, the earlier findings apply to rating terms that relate to the global structure (or artistic composition) of the images (i.e., *ordered* and *harmonious*). The rating term *pleasing*, which reflects the subjective preferences of the beholder, was the least stable of the four terms used in our study. Moreover, rating scores for *pleasing* were previously found to become higher with increasing exposure times (P. Locher et al., 2007). Together, these results support the notion that the aesthetic evaluation of artworks begins with the rapid bottom-up generation of a gist reaction (Cupchik, Vartanian, Crawley, & Mikulis, 2009; P. Locher et al., 2007; Verhavert et al., 2018). This gist reaction may be followed by a more detailed exploration of pictorial detail, which is directed in a top-down fashion by cognition-based mechanisms, provided that cognitive information is relevant in the context of viewing the artwork.

### Limitations of the Experimental Design

The present approach to enhance perceptive mechanisms has the drawback that our results are limited to a restricted set of abstract artworks, which are grayscale and represent a distinct style by a single artist. Abstract and representational art are processed differently in the brain, as they activate different sets of brain regions (Lengger, Fischmeister, Leder, & Bauer, 2007; Vartanian & Goel, 2004). Moreover, participants viewed the stimuli under laboratory conditions on a screen and not in an environment that is more seductive for aesthetic contemplation. Consequently, it remains to be established whether similar results can also be obtained for other types of art and under other circumstances, for example, for representational art on display in a museum. However, due to the cognitive overload, which prevails in such situations (Brieber, Nadal, & Leder, 2015; Specker, Tinio, & van Elk, 2017), it might be difficult to study perceptual mechanisms in isolation.

### Implications for Modeling Aesthetic Experience

The present results are relevant for current models of aesthetic experience. On the one hand, hierarchical models of aesthetic experience (Bullot & Reber, 2013; Graf & Landwehr, 2015; Leder et al., 2004; Pearce et al., 2016; Pelowski et al., 2017) postulate that perception of basic image properties (luminance, contrast, colors, spatial frequency spectrum, etc.) occurs at lower levels of visual processing, followed by cognitive mastering of explicit information about artistic style, content and context at higher levels, which eventually lead to an aesthetic experience (see “Introduction” section). In these hierarchical models, perceptual processing alone cannot culminate in an aesthetic judgment. The third component in many models of aesthetic experience, on which we did not focus in the present work, are the emotions or affective experiences provoked by an artwork (Chatterjee & Vartanian, 2014; Graf & Landwehr, 2015; Leder et al., 2004; Markovic, 2012; Redies, 2015; Silvia, 2014).

On the other hand, it has been proposed (Redies, 2015) that perceptual processing of artistic image composition (Dowling, 2014) can take place *in parallel to* and detached of cognitive processing of image content and context. In this model, artistic image composition is described as a particular arrangement of pictorial elements in the image that satisfies the artist’s aesthetic criteria. It has been postulated that other humans share these criteria and that they are largely independent of cultural context or depicted image content (Arnheim, 1954; Bell, 1914; Kandinsky, 1912; P. J. Locher et al., 1999; Malevich, 1927). In this model (Redies, 2015), both (cognitive and perceptual) processing channels must fulfill specific conditions to eventually lead to an aesthetic experience. One specific hypothesis derived from this model is that successful processing in one of the two channels can lead to an aesthetic judgment when processing in the other channel is diminished or absent. The present results provide support for this hypothesis because they suggest that stable and automatic aesthetic evaluations of image composition are possible under conditions when cognitive processing is efficiently reduced or absent.

## Author note

Claudia Menzel is now at Department of Psychology, University of Koblenz-Landau, Germany. Caitlin Mullin is now at Computational Perception and Cognition Group, Computer Science & Artificial Intelligence Laboratory, Massachusetts Institute of Technology, Cambridge, MA, USA.
